# The transcription factor TSHZ3 promotes tumor immunosuppression and inhibits metastasis in lung adenocarcinoma

**DOI:** 10.3389/fimmu.2025.1519815

**Published:** 2025-04-08

**Authors:** Xi Zhang, Yan Liu, Bai-Zhao Peng, Xing-hong Zhou, Yan-ting You, Ying Yang, Shuai Ji, Tian-yu Zhong, Xiao-hu Chen, Yan-yan Liu, Xiao-shan Zhao

**Affiliations:** ^1^ Guizhou University of Traditional Chinese Medicine, Guiyang, Guizhou, China; ^2^ School of Chinese Medicine, Southern Medical University, Guangzhou, Guangdong, China; ^3^ The Affiliated TCM Hospital of Guangzhou Medical University, Guangzhou, Guangdong, China

**Keywords:** Teashirt zinc finger homeobox 3, tumor immunity, apoptosis, lung adenocarcinoma, metastasis, epithelial-mesenchymal transition

## Abstract

Teashirt zinc finger homeobox 3 (TSHZ3) is a transcription factor implicated in the progression of certain cancers. However, its expression and function in lung adenocarcinoma (LUAD) remain unclear. Therefore, we aimed to investigate TSHZ3 expression and assess its prognostic significance in LUAD patients. First, we explored prognostic data and predicted the function of TSHZ3 in lung cancer through bioinformatics analysis. We then validated the functions using cellular and animal experiments. Our results indicated that TSHZ3 expression was significantly lower in LUAD compared to normal lung tissues. High TSHZ3 expression was positively associated with better overall survival in LUAD patients. GO and pathway analyses suggested that TSHZ3 is involved in immune responses and various cancer-related processes. Immune infiltration analysis revealed correlations between TSHZ3 and immune cell infiltration, particularly macrophages, as well as the expression of numerous immune stimulators, chemokines, and receptors. Our experiment results suggest that TSHZ3 overexpression inhibits cell migration, invasion, and epithelial–mesenchymal transition (EMT) *in vivo* and *in vitro*. LUAD cells overexpressing TSHZ3 were more prone to apoptosis due to the recruitment of CD86+ macrophages. In addition, CCL2 expression was significantly higher in LUAD cells overexpressing TSHZ3, while CCR2 expression was also significantly upregulated in co-cultured macrophages. These findings suggest that TSHZ3 is an important tumor suppressor by inhibiting EMT and metastasis while inducing apoptosis through M1 macrophage chemotaxis via the CCL2/CCR2.

## Introduction

Lung carcinoma is the leading cause of cancer-related mortality worldwide (1). As one of the main subtypes of lung cancer, lung adenocarcinoma (LUAD) has an estimated annual incidence of 1.8 million cases and an extremely poor 5-year survival rate (2, 3). However, growing evidence indicates targeting gene aberrations with personalized immunotherapy has significantly improved the survival of LUAD patients (4, 5). Therefore, identifying accurate and sensitive immune-related biomarkers is crucial for improving LUAD patient outcomes. A transcription factor is a protein that activates or inhibits gene transcription by binding to the DNA helix (6). Transcription factors play crucial roles in the development and regulation of cancer cells (7). Some transcription factors serve as key regulators in tumor immunology by inducing immunosuppression and modulating chemokines to recruit T lymphocytes (8–10). A recent study confirmed that the transcription factor RUNX3 promotes CD8+ T lymphocyte recruitment through the chemokines CCL3 and CCL20 in the immune microenvironment of LUAD (11). Moreover, transcription factors regulate the tumor immune microenvironment by influencing immune cell infiltration, including dendritic cells, neutrophils, and macrophages (12–14).

Teashirt zinc finger homeobox 3 (TSHZ3) is a transcription factor and has been widely studied in nervous system development and smooth muscle cell differentiation (15–17). TSHZ3 functions as a tumor suppressor by inhibiting cancer cell invasion in brain glioma (18). Additionally, TSHZ3 suppresses colorectal cancer through a mechanism related to DNA methylation (19). However, its role in LUAD growth and the underlying mechanism of action remain unknown. In this study, we investigated the transcriptional and protein levels of TSHZ3 in LUAD using Tumor Immune Estimation Resource (TIMER), Gene Expression Profiling Interactive Analysis (GEPIA), Oncomine, The University of ALabama at Birmingham Cancer data analysis Portal (UALCAN) database, and Human Protein Atlas (HPA). Furthermore, we predicted the potential functions of TSHZ3 in LUAD and evaluated the correlation between TSHZ3 expression and prognosis in malignancy patients using the Kaplan–Meier plotter. Lastly, we validated the functions of TSHZ3 in LUAD through cellular and animal experiments. Our findings highlight TSHZ3 as a novel and promising target for LUAD diagnosis and immunotherapy.

## Methods

### Analysis of TSHZ3 transcriptional levels

The transcriptional level of TSHZ3 across different types of cancers was preliminarily evaluated using TIMER (https://cistrome.shinyapps.io/timer/). Further analysis of TSHZ3 transcriptional levels in lung cancer was conducted via GEPIA (http://gepia.cancer-pku.cn/) (33, 34). Additionally, lung cancer subtypes with different TSHZ3 expression levels were identified using the Oncomine database (https://www.oncomine.org/resource/login.html) (35). In Oncomine, RNA data were included in a meta-analysis using the Compare module to provide high-grade evidence. A *p*-value of < 0.05 was considered statistically significant.

### Analysis of TSHZ3 protein levels

We utilized the UALCAN database (http://ualcan.path.uab.edu/) to examine the protein levels of TSHZ3 in LUAD using *Z*-score analysis (36). In addition, we validated TSHZ3 immunohistochemistry in LUAD using the HPA database (http://www.proteinatlas.org). Immunohistochemistry images were analyzed with ImageJ software, and the average optical density (AOD) was used to quantify protein levels.

### Survival analysis

We assessed the impact of TSHZ3 on clinical prognosis using the Kaplan–Meier plotter as of 8 March 2021. The Kaplan–Meier plotter (http://kmplot.com/analysis/) analyzes the effect of specific genes on survival using data from over 10,000 cancer samples, including breast, gastric, lung, and ovarian cancer samples (37). The log-rank *p*-value and HR with 95% confidence intervals were reported.

### Prediction of TSHZ3 functions

We identified co-expressed genes of TSHZ3 in LUAD using the LinkedOmics database (http://www.linkedomics.org/login.php) (38). The functions of TSHZ3 and its significant correlates were then predicted through GO enrichment and KEGG pathway analyses using the Database for Annotation, Visualization, and Integrated Discovery (DAVID) (https://david.ncifcrf.gov/summaryjsp). GO analysis predicted the function of TSHZ3 based on biological processes, cellular components, and molecular functions. KEGG pathway analysis identified pathways associated with the functions of TSHZ3.

### Immune infiltration analysis

TIMER is a comprehensive web resource for systematically evaluating immune infiltration across different cancer types (33). It estimates the abundance of immune cells from gene expression profiles using a published deconvolution-based statistical method (39). Accordingly, we first evaluated the abundance of immune infiltrates, including B cells, CD8+ T cells, CD4+ T cells, dendritic cells, neutrophils, and macrophages. Next, we further confirmed the correlations between TSHZ3 expression and immune stimulators, receptors, and chemokines using the TISDB database (http://cis.hku.hk/TISIDB) (40). The six most strongly correlated immune infiltrates of each type from the heatmap were listed. The correlation coefficient was determined using the Spearman method.

### Lentiviral infection screening and establishment of a human lung cancer A549 cell line stably expressing the TSHZ3 fusion protein

When the growth density of human lung cancer A549 cells reached 70%, the cells were infected overnight with a green fluorescent protein (GFP)-tagged lentivirus plasmid packaging TSHZ3 or negative control, purchased from GeneCopoeia (Guangzhou, China), at an MOI of 5 in serum-free medium. Simultaneously, 6 μg/ml polybrene (Sigma-Aldrich, St. Louis, MO, USA) was added to enhance lentivirus infection efficiency. The medium was replaced with fresh complete medium every 2 days, and GFP expression was analyzed using a fluorescence microscope (Olympus, Shinjuku, Tokyo, Japan) after 3 days of transduction. Meanwhile, TSHZ3 expression was measured by RT-PCR after transduction and subsequent culture with 2 µg/ml puromycin for 7 days. The A549 cells transfected with GFP-LV-vector or GFP-LV-TSHZ3 plasmids were then cultured in a medium containing 0.5 µg/ml puromycin and designated A549-TSHZ3-NC or A549-TSHZ3, respectively.

### Cell proliferation, migration, and invasion assays

A549-TSHZ3-NC and A549-TSHZ3 cells were seeded in 96-well culture plates at a density of 10^4^ cells/well, and proliferation was assessed using CCK8 (APExBIO, Houston, USA) at 24, 48, and 72 h. For the cell invasion/migration experiment, A549-TSHZ3-NC and A549-TSHZ3 cells were resuspended in serum-free RPMI 1640 medium and seeded in transwell cell culture chamber filters, which were coated on the upper side with/without Matrigel (Corning Incorporated, New York, USA). The inserts were placed in wells containing RPMI 1640 medium with 10% fetal bovine serum. After 24 h, the inserts were removed, washed with phosphate-buffered saline, fixed, and stained with crystal violet (0.05% w/v in methanol). The bottom surfaces of the stained inserts were then observed under a light microscope. Additionally, cells were seeded in six-well culture plates at a density of 5 × 10^5^ cells/well, and a scratch was created using a pipette tip. Cell migration was observed at 0 and 48 h.

### Co-culture of cancer cells with macrophages

THP-1 cells were cultured in RPMI 1640 medium supplemented with 10% fetal bovine serum and 1% penicillin–streptomycin and stimulated with 100 ng/ml phorbol 12-myristate-13-acetate (PMA) for 48 h to induce THP-1 macrophages. Human peripheral blood mononuclear cells (FH-H073), purchased from FuHeng Biology (Shanghai, China), were cultured in RPMI 1640 medium supplemented with 10% fetal bovine serum and 1% penicillin–streptomycin for 6 days to obtain macrophages. Indirect contact co-culture was performed in 24-well plates using 8 μm polyethylene terephthalate membrane filters (Corning, USA). In brief, the macrophages (5 × 10^4^) cells) were seeded in the upper chambers, while A549-TSHZ3-NC or A549-TSHZ3 cells (1.5 × 10^5^ cells) were seeded in the lower chambers. After 24 h, the bottom surfaces of macrophages were observed, and cancer cells in the lower chamber were collected for apoptosis analysis. In addition, A549-TSHZ3-NC and A549-TSHZ3 cells were plated in 100 mm dishes containing basic RPMI 1640 medium supplemented with 10% fetal bovine serum (FBS). Once cell confluence reached 80%–90%, the medium was collected and centrifuged at 1,500 rpm for 5 min. The supernatant was then used to culture macrophages, after which CD86 and CCR2 expression were detected.

### Flow cytometry analysis

The macrophage cells from the co-culture system were harvested and stained with PE-conjugated anti-CD86 antibody (eBioscience, Waltham, USA) or Allophycocyanin (APC)-conjugated anti-CCR2 antibody (Biolegend, San Diego, USA) in PBS for 30 min in the dark. The stained cells were then analyzed by flow cytometry. Similarly, A549-TSHZ3-NC or A549-TSHZ3 cells from the co-culture system were harvested, and apoptotic cells were measured by Annexin V APC/PI (MultiSciences, Hangzhou, China).

### Fluorescence monitoring

Fluorescence microscopy analysis of A549-TSHZ3-NC and A549-TSHZ3 cells was conducted using a microscope (Olympus, Shinjuku, Tokyo, Japan) equipped with a GFP-specific filter.

### Quantitative real-time polymerase chain reaction

Total RNA was extracted from A549-TSHZ3-NC and A549-TSHZ3 cells, and the first-strand cDNA was synthesized using a reagent kit purchased from TransGen Biotech (Beijing, China). qPCR was performed using the SYBR Premix Ex Taq II reaction system. Real-time PCR was conducted with TransStart Tip Green qPCR SuperMix (TransGene Biotech, Beijing, China). The expression of target genes was normalized to the housekeeping gene Glyceraldehyde-3-Phosphate Dehydrogenase (GAPDH). The primer sequences used in the experiment are shown below: GAPDH (Forward 5′-AGAAGGCTGG GGCTCATTTG-3′, Reverse 5′-AGGGGCCATCCACAGTCTTC-3′), TSHZ3 (Forward 5′-CAGAGGAGCATACGGCAGAT-3′, Reverse 5′-TGTGTGACTCGCTGTCC ATT-3′), and CCL2 (Forward 5′-CAGCCAGATGCAATCAATGCC-3′, Reverse 5′-TGG AATCCTGAACCCACTTCT-3′).

### Western blotting

Cells were harvested and lysed with lysate (Beyotime, Shanghai, China) supplemented with a protease inhibitor cocktail (Beyotime, China). The protein aliquots were separated on 10% sodium dodecyl sulfate-polyacrylamide gel (SDS-PAGE), transferred onto PVDF membranes (Millipore, Darmstadt, Germany), and blocked with 5% bovine serum albumin (BSA). The membranes were then incubated with the indicated primary antibodies overnight at 4°C, followed by the corresponding secondary antibodies for 1 h at room temperature. Protein detection was performed using enhanced chemiluminescence (Thermo Fisher, Carlsbad, CA, USA) according to the manufacturer’s protocol.

### Mouse experiments

Male BALB/c nude mice (4 weeks old) were purchased from Guangdong Medical Laboratory Animal Center and maintained in a specific pathogen-free environment at 25°C under a 12-h light/dark cycle. The nude mice were injected with 1 × 10^6^ A549 cells stably overexpressing TSHZ3 or control vectors in 0.1 ml of phosphate-buffered saline via the lateral tail vein (*n* =  5 per group). After 4 weeks, all mice were killed, and GFP intensity in lung, liver, and spleen tissues was detected using multimodal imaging (*In-Vivo* FX PRO, Bruker, Ettlingen, Germany). The lung and liver tissues were dissected and fixed in 10% formalin for hematoxylin and eosin staining. The animal experiment was approved by the Standards for Animal Ethics in the Guangdong Medical Laboratory Animal Center (C202205-12) and conducted in accordance with relevant guidelines and regulations for the care and use of experimental animals.

### Statistical analysis

We presented continuous data as mean ± standard deviation (SD) and analyzed them using the Student’s *t*-test in GraphPad Prism (version 8.0).

## Discussion

The transcription factor TSHZ3 is essential for the development of neural circuitry that controls breathing (20). TSHZ3 plays distinct roles in different cancer types. Here, we found that the transcriptional level of TSHZ3 is downregulated in several solid cancers, including LUAD, compared with normal tissue, consistent with previous studies (21, 22). However, no significant difference was observed between LUSC and normal tissue in TSHZ3 transcription, suggesting a specific role of TSHZ3 in the carcinogenesis and progression of LUAD. We further analyzed the protein level of TSHZ3 and found a significant reduction in LUAD compared with normal lung tissues. These findings support TSHZ3 as a potential diagnostic biomarker and tumor suppressor for LUAD, warranting further investigation.

As a potential tumor suppressor, TSHZ3 may influence the prognosis of patients with LUAD. Therefore, we assessed its role in LUAD prognosis and found that high TSHZ3 expression predicts a favorable outcome, reinforcing our hypothesis that TSHZ3 acts as a tumor suppressor in this cancer. Numerous studies have reported that N-cadherin and vimentin are downregulated, while E-cadherin is upregulated during the EMT process of LUAD (23–25). Our *in vitro* experiments align with these findings, demonstrating that TSHZ3 overexpression in LUAD cells suppresses N-cadherin and vimentin expression while promoting E-cadherin expression. Additionally, we confirm that TSHZ3 overexpression inhibits LUAD cell migration and invasion. Furthermore, our mouse experiments validate that TSHZ3 overexpression reduces distant metastasis in LUAD. Finally, we observed a significant increase in the apoptosis rate of A549 cells overexpressing TSHZ3 when co-cultured with macrophages. These findings supported TSHZ3 as a prognostic biomarker in LUAD.

We performed GO enrichment analysis and KEGG pathway analysis on TSHZ3 and its co-expressed genes to investigate the underlying antitumor mechanism of TSHZ3. Significant enrichment was observed in cellular components related to the inflammatory response, immune response, and cell adhesion. Additionally, KEGG pathway analysis identified pathways involved in tumorigenesis and the pathogenesis of LUAD, including pathways in cancer, proteoglycans in cancer, the PI3K-Akt signaling pathway, cytokine–cytokine receptor interaction, the MAPK signaling pathway, the chemokine signaling pathway, and endocytosis. These findings suggest that immune infiltration is likely a key factor in the antitumor effects of TSHZ3. We investigated the relationship between immune infiltration and TSHZ3 expression and found that TSHZ3 is positively correlated with the infiltration of various immune cells, including macrophages. Furthermore, TSHZ3 expression in LUAD tissue is positively correlated with the immunostimulator CD86, the chemokine C-C Motif Chemokine Ligand (CCL), and the receptor C-C Motif Chemokine Receptor (CCR2). CD86 serves as a surface marker of M1 macrophages (26). CCL2 plays a crucial role in stimulating host antitumor activity and acts as a potent chemokine for monocytes, memory T lymphocytes, and natural killer (NK) cells (27, 28).

We validated these findings again in subsequent experiments. When A549 cells were not co-cultured with macrophages, TSHZ3 overexpression had no effect on cell viability or apoptosis. However, when co-cultured with macrophages, LUAD cells overexpressing TSHZ3 were more prone to apoptosis due to the recruitment of CD86+ macrophages. In the tumor microenvironment, macrophages can differentiate into two major phenotypes: M1 macrophages (tumor-suppressing subtype) and M2 macrophages (tumor-promoting subtype) (29–31). Co-culturing macrophages with lung cancer cell lines can upregulate CCR2/CCL2 and CX3CR1/CX3CL1 in both cancer cells and macrophages, playing a central role in lung cancer growth and metastasis (32). Consistent with this study, we found that CCL expression was significantly higher in LUAD cells overexpressing TSHZ3, while CCR2 expression was significantly upregulated in co-cultured macrophages. These findings suggest that TSHZ3 overexpression in LUAD cells induces apoptosis by recruiting M1 macrophages via the CCL2/CCR2 axis.

Overall, our study demonstrates that TSHZ3 expression is reduced in LUAD. Furthermore, TSHZ3 overexpression in LUAD is associated with a favorable prognosis and inhibits EMT and metastasis. Finally, overexpressing TSHZ3 in LUAD cells induces apoptosis through M1 macrophage chemotaxis via the CCL2/CCR2.

## Results

### The transcriptional and protein levels of TSHZ3 are decreased in LUAD

**Figure 1:**
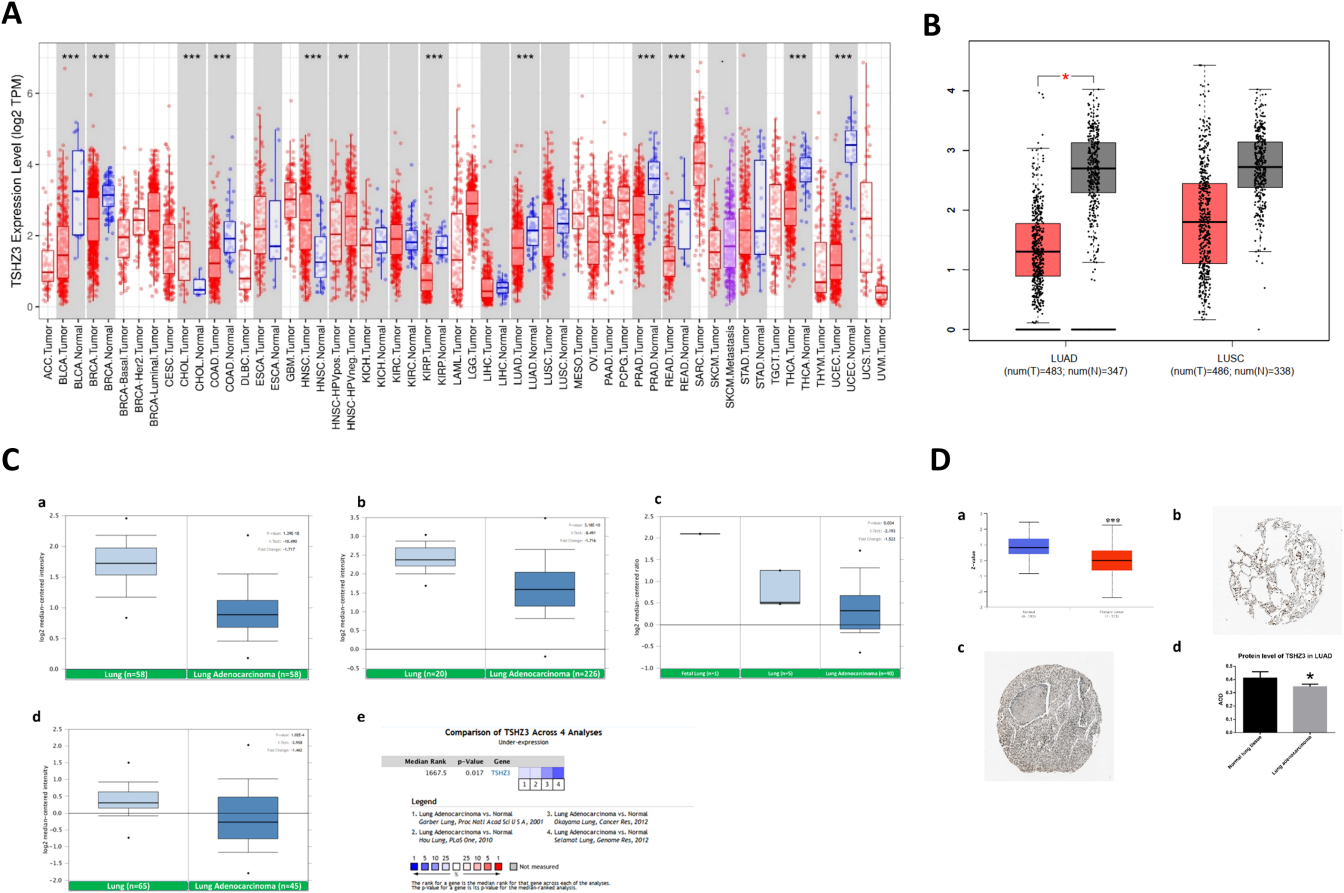
The expression level of TSHZ3 in TIMER, GEPIA, Oncomine, and HPA. **(A)** Transcription levels of TSHZ3 across different cancer types (TIMER). **(B)** Transcription levels of TSHZ3 in lung cancer (GEPIA). ^*^ *p* < 0.05; ^**^ *p* < 0.01; ^***^ *p* < 0.001. **(C)** Transcription levels of TSHZ3 in lung cancer in Oncomine. (a–d) TSHZ3 is downregulated in lung adenocarcinoma in the Selamat lung, Okayama lung, Garber lung, and Hou lung. (e) Meta-analysis suggests that TSHZ3 is downregulated in lung adenocarcinoma. **(D)** Protein levels of TSHZ3 in LUAD and normal lung tissue based on HPA. (a) Protein levels of TSHZ3 in LUAD and normal lung tissue based on UALCAN. (b) Protein levels of TSHZ3 in normal lung tissue based on HPA (both alveolar cells and macrophages were stained) (staining: high; intensity: strong; quantity: > 75%). (c) Protein levels of TSHZ3 in LUAD based on HPA (staining: low; intensity: weak; quantity: > 75%). (d) Protein levels of TSHZ3 between LUAD and normal lung tissue based on HPA. ^*^ *p* < 0.05; ^**^ *p* < 0.01; ^***^ *p* < 0.001. ACC, adrenocortical carcinoma; BLCA, bladder urothelial carcinoma; BRCA, breast invasive carcinoma; CESC, cervical squamous cell carcinoma and endocervical adenocarcinoma; CHOL, cholangiocarcinoma; COAD, colon adenocarcinoma; DLBC, lymphoid neoplasm diffuse large B-cell lymphoma; ESCA, esophageal carcinoma; GBM, glioblastoma multiforme; HNSC, head and neck squamous cell carcinoma; KICH, kidney chromophobe; KIRC, kidney renal clear cell carcinoma; KIRP, kidney renal papillary cell carcinoma; LAML, acute myeloid leukemia; LGG, brain lower-grade glioma; LIHC, liver hepatocellular carcinoma; LUAD, lung adenocarcinoma; LUSC, lung squamous cell carcinoma; MESO, mesothelioma; OV, ovarian serous cyLUADenocarcinoma; PAAD, pancreatic adenocarcinoma; PCPG, pheochromocytoma and paraganglioma; PRAD, prostate adenocarcinoma; READ, rectum adenocarcinoma; SARC, sarcoma; SKCM, skin cutaneous melanoma; STAD, stomach adenocarcinoma; TGCT, testicular germ cell tumors; THCA, thyroid carcinoma; THYM, thymoma; UCEC, uterine corpus endometrial carcinoma; UCS, uterine carcinosarcoma; UVM, uveal melanoma.

The analysis revealed that TSHZ3 transcriptional levels were lower in nine cancer types, including LUAD, but higher in cholangiocarcinoma (CHOL) and head and neck squamous cell carcinoma (HNSC) compared with the normal tissues (**Figure 1A**). We further validated TSHZ3 transcriptional levels in LUAD using two additional public databases (GEPIA and Oncomine). Both the results suggested the transcriptional levels of TSHZ3 were downregulated significantly in LUAD compared with normal lung tissues (**Figures 1B, C**). Data from UALCAN revealed that TSHZ3 protein levels were significantly reduced in LUAD compared with normal lung tissues. Immunohistochemistry results also indicated that TSHZ3 protein levels were significantly lower in LUAD (*n* = 6) than in normal lung tissues (*n* = 3) (**Figure 1D**). Interestingly, macrophages were also highly stained, in addition to alveolar cells, in normal lung tissues.

### The high expression level of TSHZ3 predicts a good prognosis in LUAD

**Figure 2:**
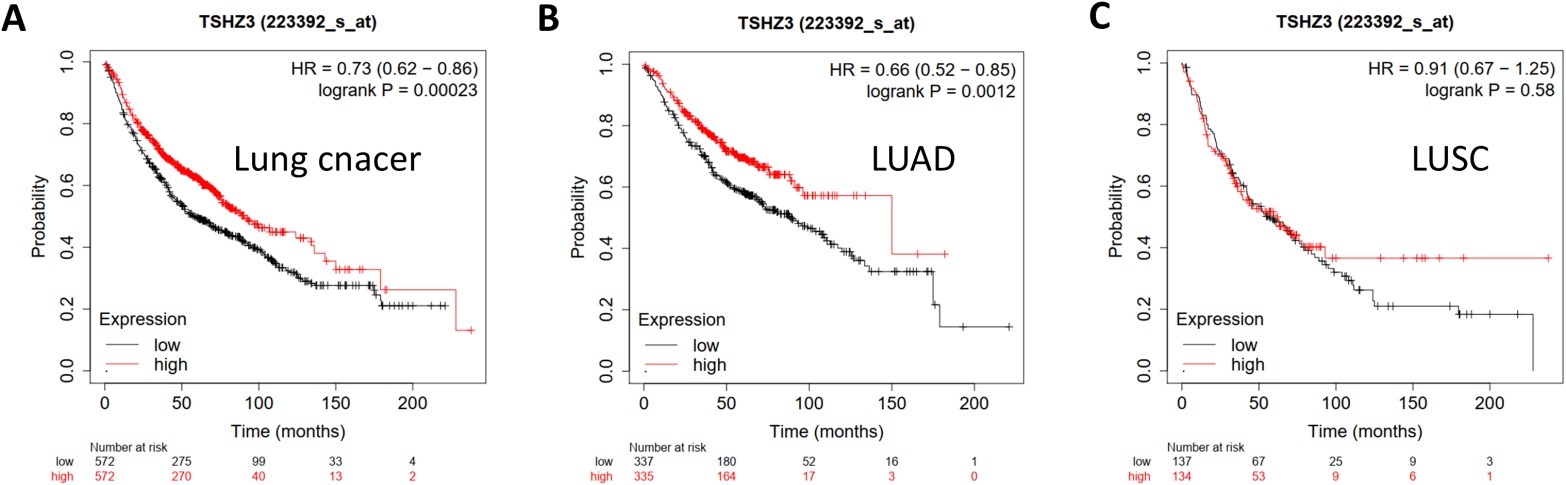
Kaplan–Meier plots comparing high and low TSHZ3 expression in lung cancer using the Kaplan–Meier plotter. **(A)** Kaplan–Meier plot of OS in lung cancer (*n* = 1,144). **(B)** Kaplan–Meier plot of OS in LUAD (*n* = 672). **(C)** Kaplan–Meier plot of OS in LUSC (*n* = 271).

We evaluated the prognostic potential of TSHZ3 in lung cancers by generating survival curves using the Kaplan–Meier plotter. The results suggested that high TSHZ3 expression levels were correlated with a favorable prognosis of overall survival (OS) in lung cancer (OS: hazards ratio (HR) = 0.73, 95% confidence intervals (CI) = 0.62 to 0.86, *p* = 0.00023) and LUAD (HR = 0.66, 95% CI = 0.52 to 0.85, *p* =0.0012). However, TSHZ3 expression levels showed no significant correlation with lung squamous cell carcinoma (LUSC) (HR = 0.91, 95% CI = 0.67 to 1.25, *p* = 0.58) (**Figure 2**).

### TSHZ3 may be involved in inflammation, immune responses, and cancer-related pathways

**Figure 3:**
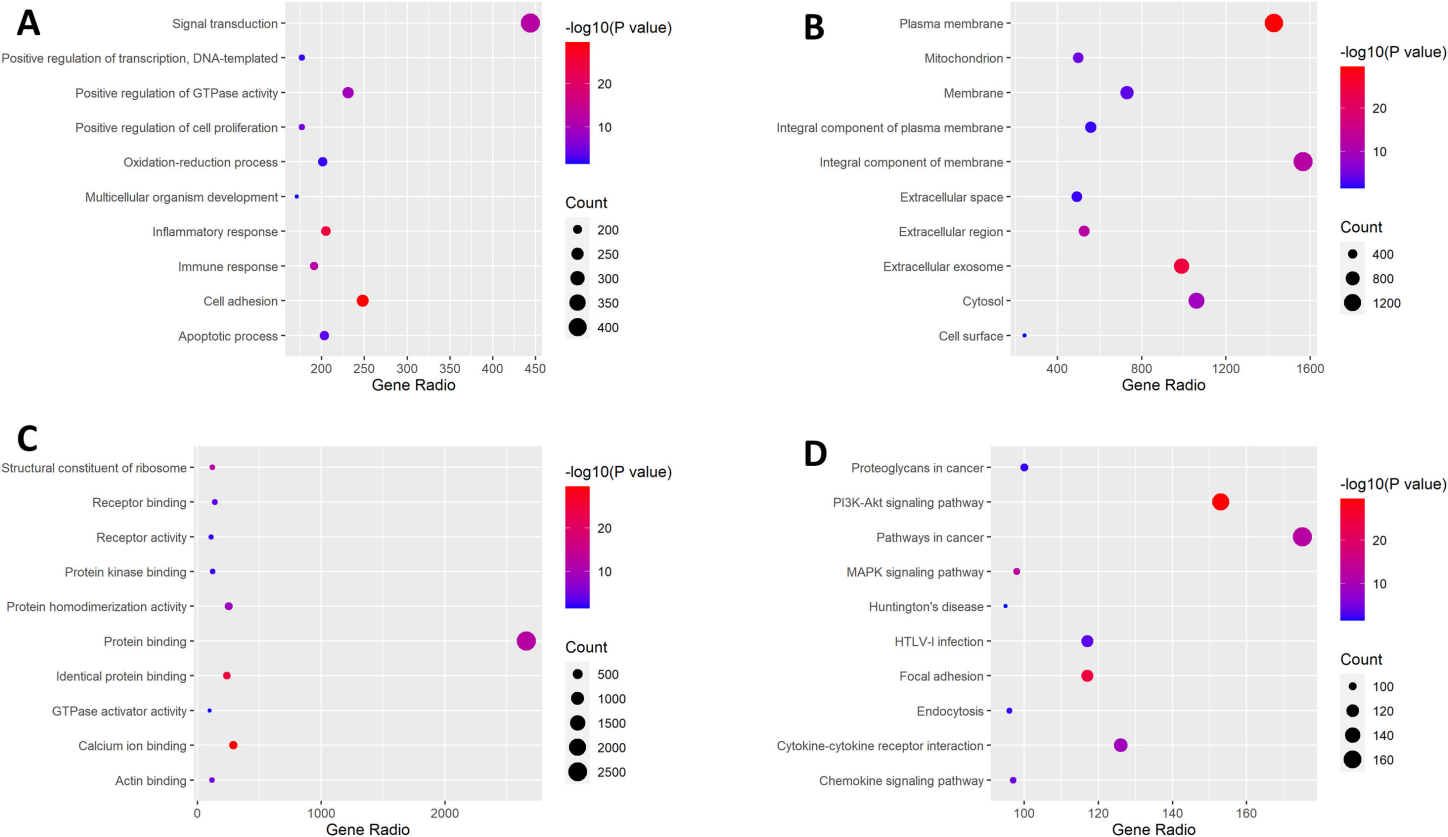
GO enrichment analysis and KEGG pathway analysis. **(A)** The top 10 significantly enriched biological processes. **(B)** The top 10 significantly enriched cellular components. **(C)** The top 10 significantly enriched molecular functions. **(D)** The top 10 significantly enriched KEGG pathway.

The top 6,000 DEGs were included in gene ontology (GO) enrichment and Kyoto Encyclopedia of Genes and Genomes (KEGG) pathway analyses (5,469 genes were identified as *Homo sapiens*). We found that the inflammatory response, immune response, and cell adhesion were significantly regulated by TSHZ3 in LUAD (**Figures 3A, B**). Among the top 10 pathways identified through KEGG pathway analysis, pathways in cancer, proteoglycans in cancer, the PI3K-Akt signaling pathway, cytokine–cytokine receptor interaction, focal adhesion, the mitogen-activated protein kinases (MAPK) signaling pathway, the chemokine signaling pathway, and endocytosis may be involved in the tumorigenesis and pathogenesis of LUAD (**Figures 3C, D**).

### TSHZ3 expression is positively correlated with immune infiltration levels in LUAD

**Figure 4:**
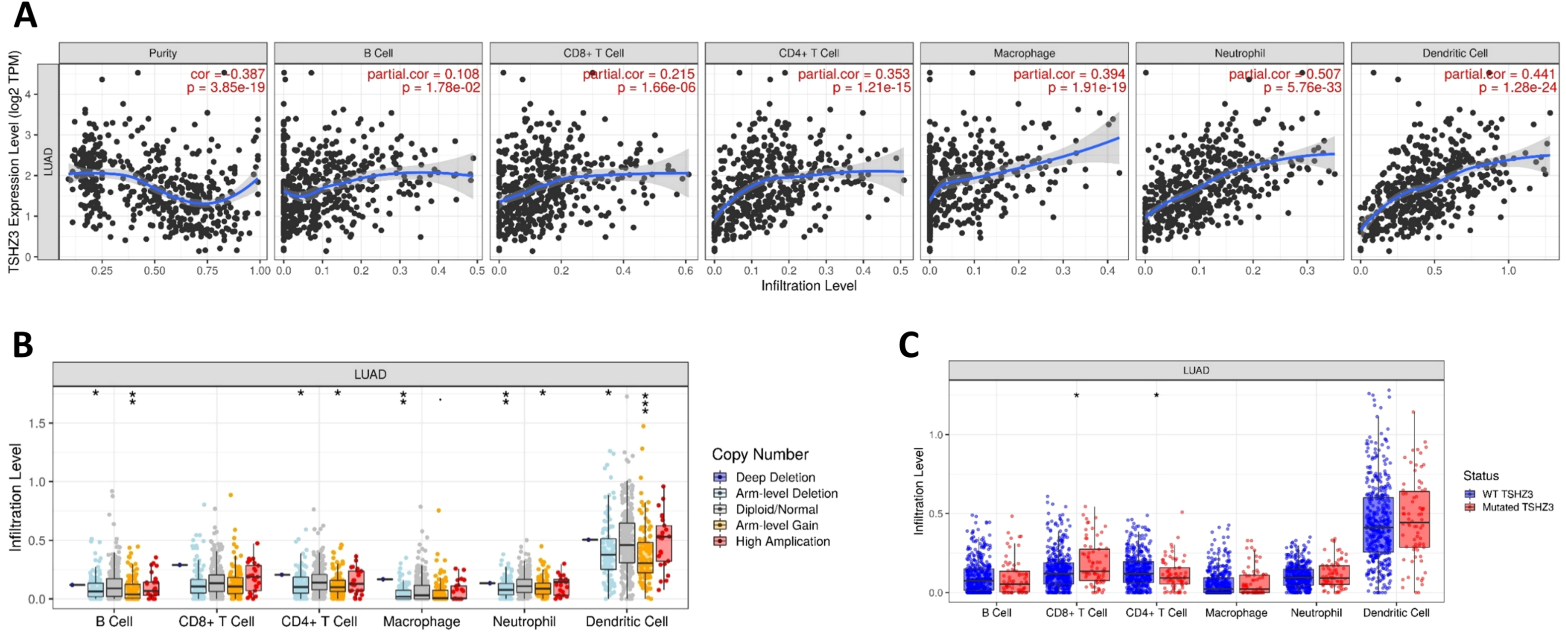
Correlation between immune infiltration level and TSHZ3 expression in LUAD. **(A)** TSHZ3 expression is significantly positively correlated with infiltrating levels of B cells, CD8+ T cells, CD4+ T cells, macrophages, neutrophils, and dendritic cells in LUAD. **(B)** Immune cell infiltration levels in LUAD across different TSHZ3 copy number variations. **(C)** Immune cell infiltration levels in LUAD individuals with wild-type or mutated TSHZ3.

We evaluated the correlations between TSHZ3 expression and immune infiltration profiles in LUAD. The results showed significant positive correlations between TSHZ3 expression levels and the infiltration levels of all listed TILs, including B cells (*r* = 0.108, *p* = 1.78*e*−02), CD8+ T cells (*r* = 0.215, *p* = 1.66*e*−06), CD4+ T cells (*r* = 0.353, *p* = 1.21*e*−15), macrophages (*r* = 0.394, *p* = 1.91*e*−19), neutrophils (*r* = 0.507, *p* = 5.76*e*−33), and dendritic cells (DCs) (*r* = 0.441, *p* = 0.28*e*−24) in LUAD (**Figure 4A**). We also investigated immune infiltration levels in LUAD across different CNA and mutation states of TSHZ3. The results indicated that the infiltration levels of B cells, CD4+ T cells, neutrophils, and DCs were significantly higher in both the arm-level deletion and arm-level gain groups compared with the diploid (normal copy number) group. Additionally, macrophage infiltration levels in LUAD were significantly elevated in the arm-level deletion group compared with the diploid group (**Figure 4B**). Furthermore, TSHZ3 mutations were associated with a higher infiltration level of CD8+ T cells and a lower infiltration level of CD4+ T cells (**Figure 4C**).

### TSHZ3 expression is positively correlated with key immune molecules in LUAD

**Figure 5:**
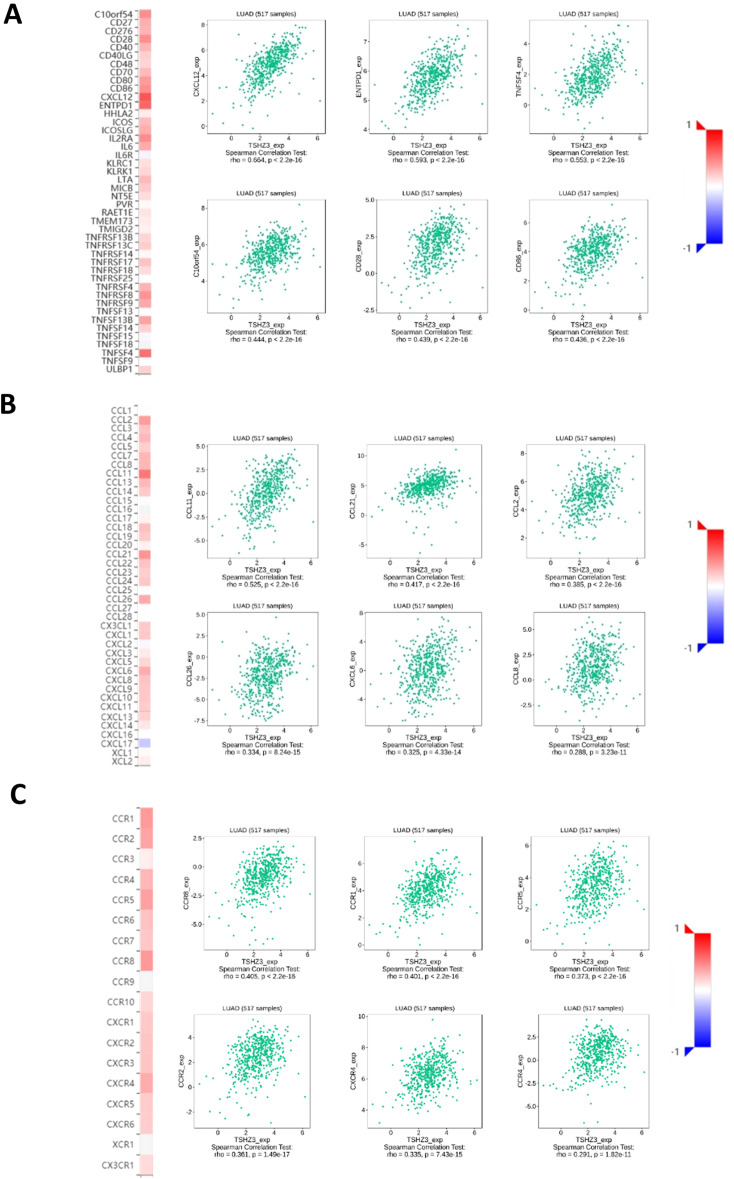
Relationships between immune molecules and TSHZ3 expression (plus the top six molecules with significant correlations). **(A)** Relationships between immune-stimulator and TSHZ3 expression. **(B)** Relationships between chemokine and TSHZ3 expression. **(C)** Relationships between receptor and TSHZ3 expression.

The heatmaps from translation initiation site database (TISDB) suggested that TSHZ3 expression is positively correlated with the expression of various immune stimulators, chemokines, and receptors in LUAD (**Figure 5**). The top six significantly associated immune stimulators are CXCL12, ENTPD1, TNFSF4, C10orf54, CD28, and CD86. The top six strongly linked chemokines are CCL11, CCL21, CCL2, CCL26, CXCL6, and CCL8. In addition, the top six significantly associated receptors are CCR8, CCR1, CCR5, CCR2, CXCR4, and CCR4.

### TSHZ3 overexpression inhibits cell migration, invasion, and EMT in A549 cells

**Figure 6:**
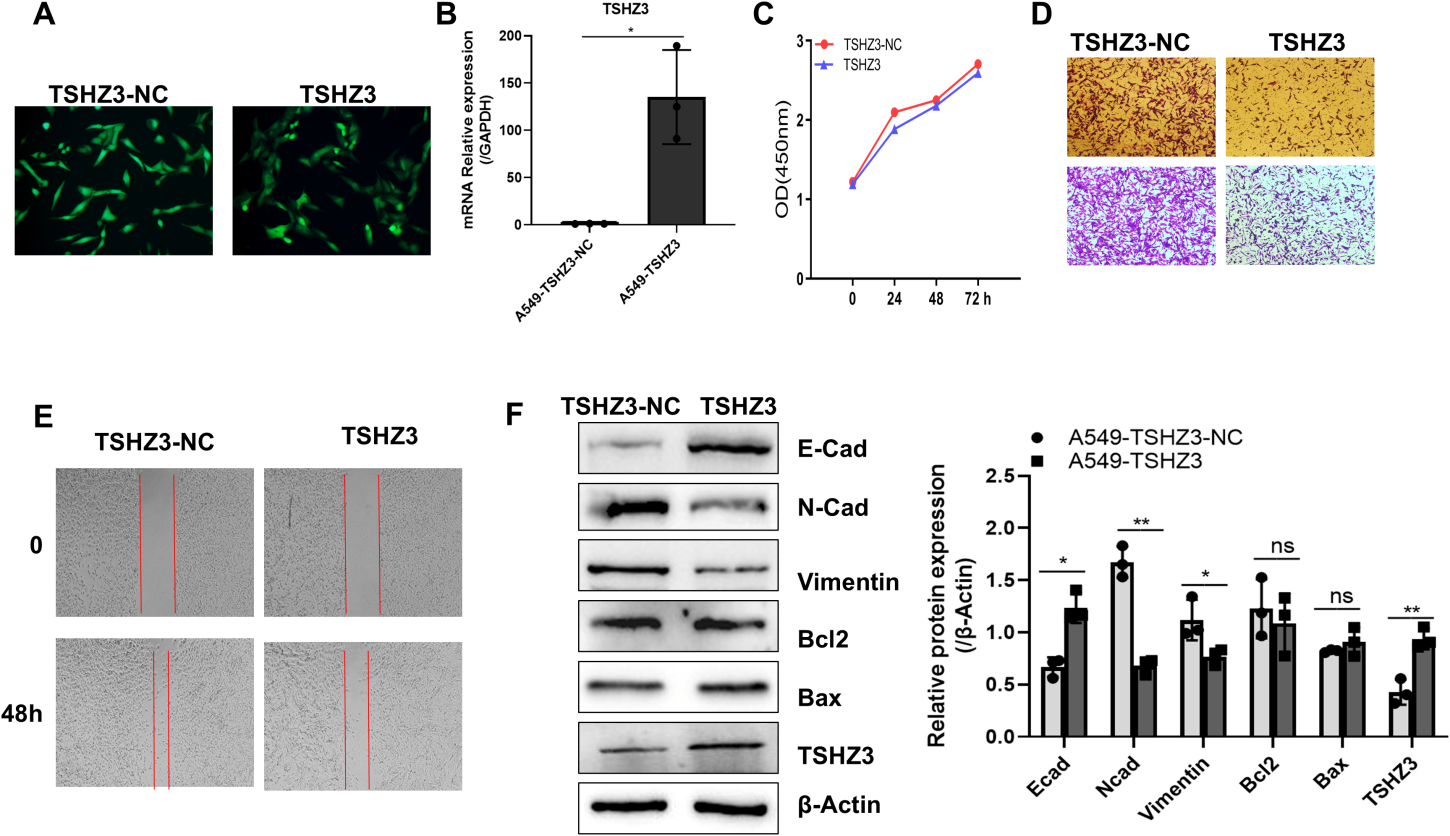
**(A)** TSHZ3-overexpressing A549 cell line was established, which inhibited cell migration, invasion, and EMT marker gene expression *in vitro*. Fluorescence microscopy was used to detect the GFP expression in A549 cells transfected with TSHZ3 or control vectors. **(B)** TSHZ3 expression was detected using real-time quantitative PCR. **(C)** The proliferation of A549-TSHZ3-NC and A549-TSHZ3 cells was measured at 0, 24, 48, and 72 h using CCK8, following the manufacturer’s instructions. **(D)** Cell invasion/migration assays were performed using a transwell cell culture chamber with A549-TSHZ3-NC and A549-TSHZ3 cells. **(E)** A wound-healing assay was conducted by creating a scratch with a pipette tip, and cell migration was observed at 0 and 48 h under a light microscope. **(F)** Western blotting was used to detect TSHZ3, EMT-related proteins (E-cadherin, N-cadherin, and vimentin), and apoptosis-related proteins (Bax and Bcl2). Experiments were performed three times.^*^ *p* < 0.05; ^**^ *p* < 0.01; ns, not significant. Data are shown as mean ± SEM.

According to our previous analysis, TSHZ3 expression is strongly negatively associated with the prognosis of LUAD. Here, we transferred A549 cells with a lentivirus-carrying DNA targeting the *TSHZ3* gene and assessed its expression using fluorescence microscopy and real-time quantitative PCR. As shown in **Figures 6A, B**, the screened A549 cells could stably express GFP and TSHZ3. After profiling TSHZ3 expression in A549 cells, we examined its effects on proliferation and metastasis. As shown in **Figures 6C–E**, TSHZ3 overexpression inhibits cell mobility and invasion in A549 but does not affect proliferation. To further validate these findings, we performed Western blotting to analyze epithelial–mesenchymal transition (EMT)- and apoptosis-related proteins. The results revealed that the epithelial marker E-cadherin was upregulated, whereas mesenchymal markers, including N-cadherin and vimentin, were downregulated. However, no significant differences were observed in the apoptosis-related proteins Bax and Bcl2 between A549 cells with stable TSHZ3 overexpression and control cells (**Figure 6F**). These data suggest that TSHZ3 overexpression may suppress the migration and invasion of A549.

### Co-culture of THP-1 macrophages with A549-TSHZ3 cells promotes CD86+ macrophage chemotaxis and cancer cell death

**Figure 7:**
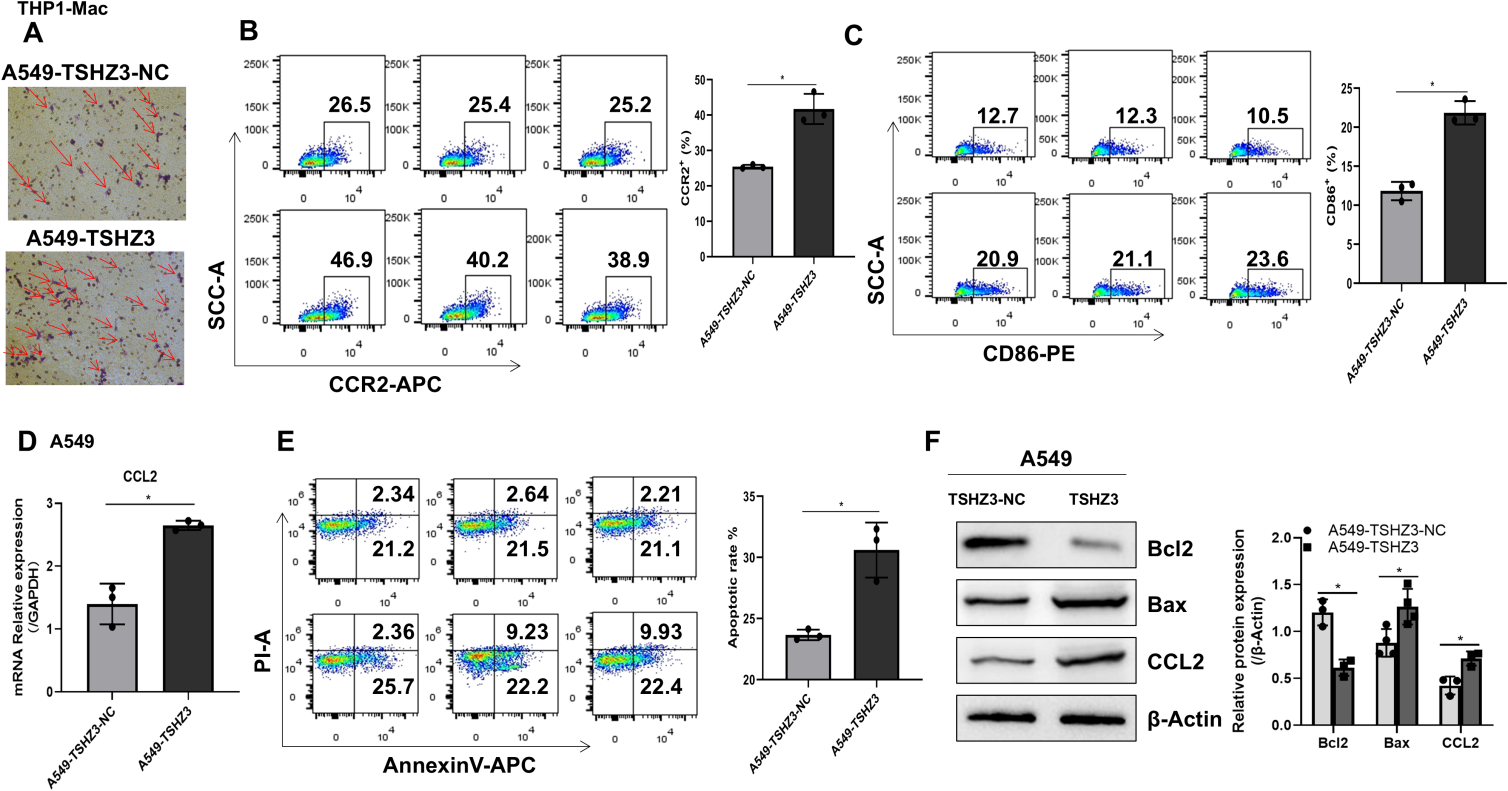
Co-culturing THP-1 macrophages with A549-TSHZ3 cells enhances macrophage chemotaxis and A549 cell apoptosis. THP-1 macrophages were co-cultured with either A549-TSHZ3-NC or A549-TSHZ3 cells, and then the bottom surfaces of THP-1 macrophages were observed. **(B**, **C)** CCR2 and CD86 expression in THP-1 macrophages were assayed using flow cytometry after co-culture. **(D)** RT-PCR was performed to assess CCL2 gene expression in A549-TSHZ3-NC and A549-TSHZ3 cells. **(E)** Apoptotic cells in A549-TSHZ3-NC and A549-TSHZ3 groups were assessed using flow cytometry. **(F)** CCL2 and apoptosis-related protein expression were analyzed via Western blotting. ^*^ *p* < 0.05; ns, not significant. Data are shown as mean ± SEM, representative of three independent experiments with similar results.

Our immune infiltration analysis revealed that TSHZ3 expression in LUAD tissues positively correlates with the infiltration of immune cells, including macrophages. To further verify these predictions, THP-1-derived macrophages were co-cultured with either A549-TSHZ3-NC or A549-TSHZ3 cells in polyethylene terephthalate membrane filters. After 24 h of co-culture, an increased number of THP-1 macrophages was observed on the bottom surface in A549 cells with stable TSHZ3 overexpression compared to control cells (**Figure 7A**). Additionally, CCR2 expression in macrophages, along with CD86+ macrophages, was significantly upregulated after co-culture with A549-TSHZ3 cells compared to the control group (**Figures 7B, C**). Importantly, the CCR2 ligand, CCL2, was also elevated in A549-TSHZ3 cells compared to A549-TSHZ3-NC cells (**Figures 7D, F**). Finally, cancer cells were collected after co-culture with THP-1 macrophages for apoptosis analysis. As shown in **Figure 7E**, a higher number of apoptotic cells was detected in A549-TSHZ3 cells than in A549-TSHZ3-NC cells, as assessed by flow cytometry. Synchronously, the apoptosis-related protein BAX was upregulated, whereas BCL2 expression was downregulated in A549-TSHZ3 cells compared to control cells (**Figure 7F**). These results suggest that THSZ3 overexpression may promote macrophage chemotaxis and A549 cell death through the CCR2/CCL2 axis. In addition, we verified these findings by co-culturing human peripheral blood mononuclear cells (PBMC) with either A549-TSHZ3-NC or A549-TSHZ3 cells, as shown in **Supplementary Figure S1**.

### TSHZ3 overexpression reduced cell migration and invasion in A549 cells *in vivo*

**Figure 8:**
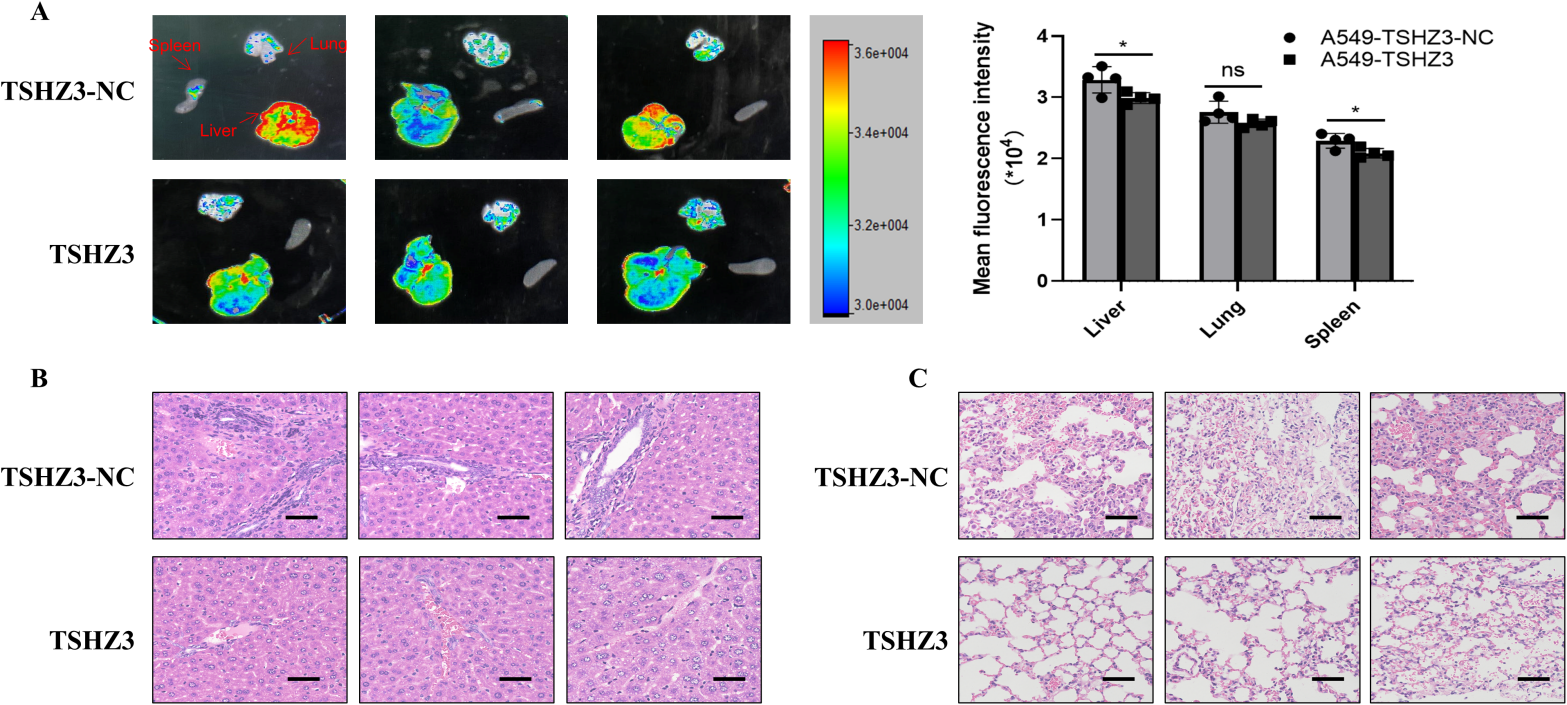
TSHZ3 over-expression reduced cell migration and invasion in A549 cells *in vivo*. The nude mice were injected with 1 × 10^6^ A549 cells stably overexpressing TSHZ3 or control vectors in 0.1 ml of phosphate-buffered saline via the lateral tail vein for 4 weeks. **(A)** The GFP intensity in lung and liver and spleen tissues were detected using Multimodal Imaging. **(B, C)** The tumor metastasis in liver and lung were detected using H&E staining. Scale bars = 100 μm. *p < 0.05; ns, non-significant.

To determine whether TSHZ3 overexpression downregulates cell mobility and invasion *in vivo*, nude mice were injected with 1 × 10^6^ A549 cells stably overexpressing TSHZ3 or control vectors in 0.1 ml of phosphate-buffered saline via the lateral tail vein and observed for 4 weeks. As shown in **Figure 8A**, GFP intensity was detected in lung, liver, and spleen tissues. The liver of control mice injected with A549-TSHZ3-NC cells exhibited significantly stronger GFP intensity than that of mice injected with A549-TSHZ3 cells. Interestingly, the GFP expression was also detected in the spleen in the group injected with A549-TSHZ3-NC cells but not A549-TSHZ3. Additionally, pathological examination of the liver and lung showed reduced numbers of metastatic nodules in the lung and less of cells with hyperchromatic nuclei in the liver after injecting A549 -TSHZ3 cells into mice than the control group (**Figures 8B, C**). It indicated that TSHZ3 may act as a tumor suppressor gene in LUAD.

## Data Availability

The original contributions presented in the study are included in the article/**Supplementary Material**, further inquiries can be directed to the corresponding author/s.

## Supplementary material

Supplementary Figure 1
**(A)** Co-culturing PBMC macrophages with A549-TSHZ3 cells promotes macrophage chemotaxis and A549 cell apoptosis. The PBMC macrophages were co-cultured with A549-TSHZ3-NC or A549-TSHZ3 cells, and then the bottom surfaces of macrophages were observed. **(B)** and **(C)** The CCR2 and CD86 expression in PBMC-macrophages were assayed using flow cytometry after co-culture. **(D)** The RT-PCR was used to detect the gene expression of CCL2 in A549-TSHZ3-NC and A549-TSHZ3 cells. **(E)** The apoptosis cells of A549-TSHZ3-NC and A549-TSHZ3 cells were measured with flow cytometry. **(F)** The CCL2 and apoptosis-related protein expression were detected using western blotting. *p<0.05,**p<0.01, ns, not significant. Data are shown as mean ± SEM representative of three independent experiments with similar results.
