# Supplementary figures and images for: The transcription factor TSHZ3 promotes tumor immunosuppression and inhibits metastasis in lung adenocarcinoma
Source: Front Immunol. 2025 Apr 8;16:1519815. doi: 10.3389/fimmu.2025.1519815 (PMC12011852; doi:10.3389/fimmu.2025.1519815)

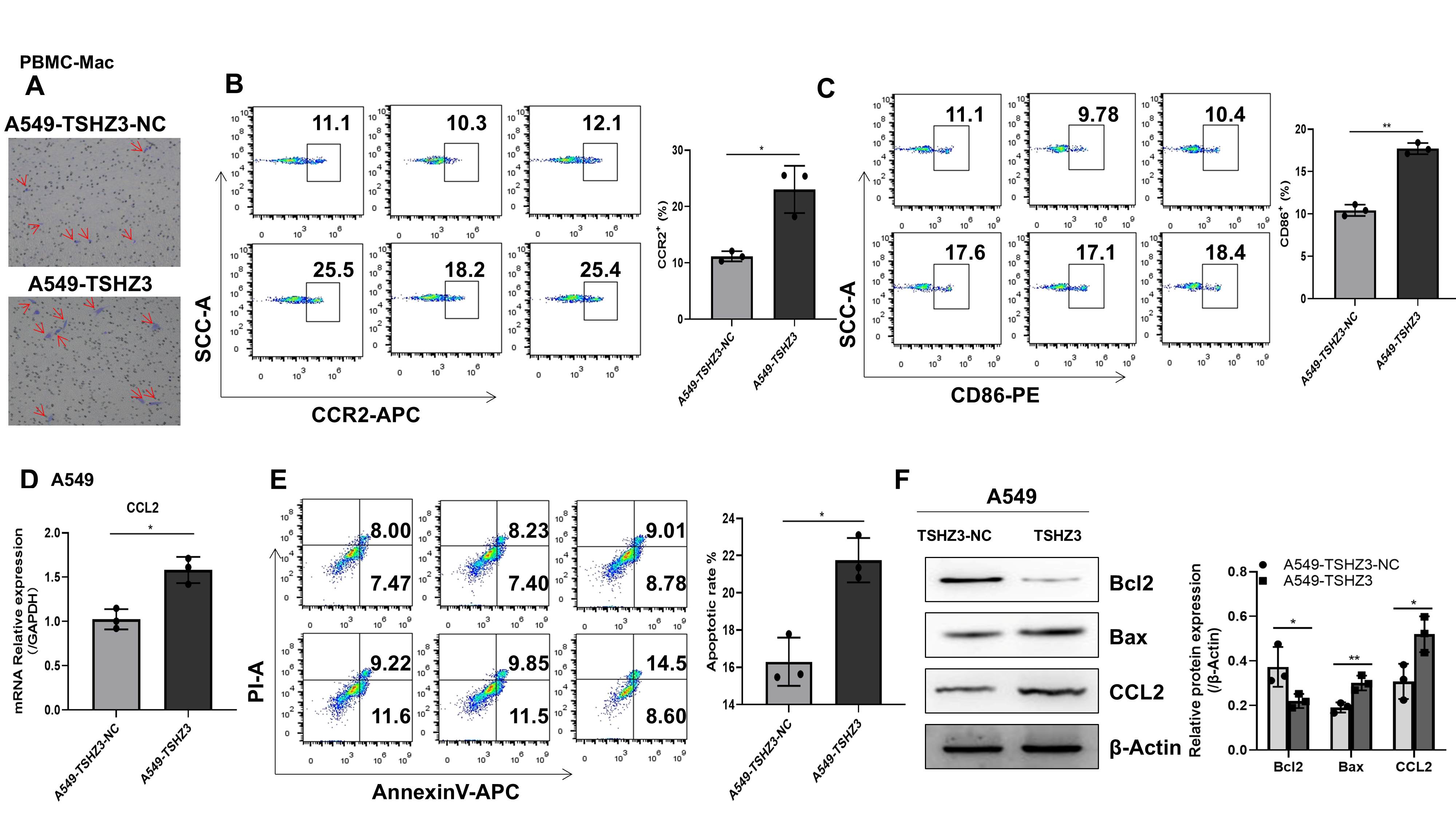

Supplement: Supplementary Figure 1 — (A) Co-culturing PBMC macrophages with A549-TSHZ3 cells promotes macrophage chemotaxis and A549 cell apoptosis. The PBMC macrophages were co-cultured with A549-TSHZ3-NC or A549-TSHZ3 cells, and then the bottom surfaces of macrophages were observed. (B) and (C) The CCR2 and CD86 expression in PBMC-macrophages were assayed using flow cytometry after co-culture. (D) The RT-PCR was used to detect the gene expression of CCL2 in A549-TSHZ3-NC and A549-TSHZ3 cells. (E) The apoptosis cells of A549-TSHZ3-NC and A549-TSHZ3 cells were measured with flow cytometry. (F) The CCL2 and apoptosis-related protein expression were detected using western blotting. *p<0.05,**p<0.01, ns, not significant. Data are shown as mean ± SEM representative of three independent experiments with similar results. [file Image1.tif]

FIGURE6F

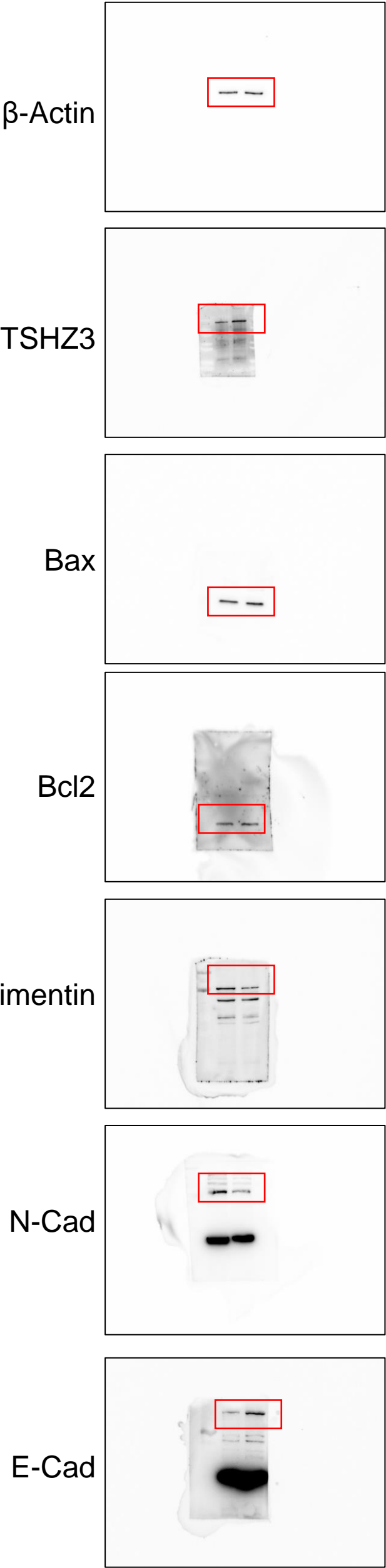

FIGURE7F

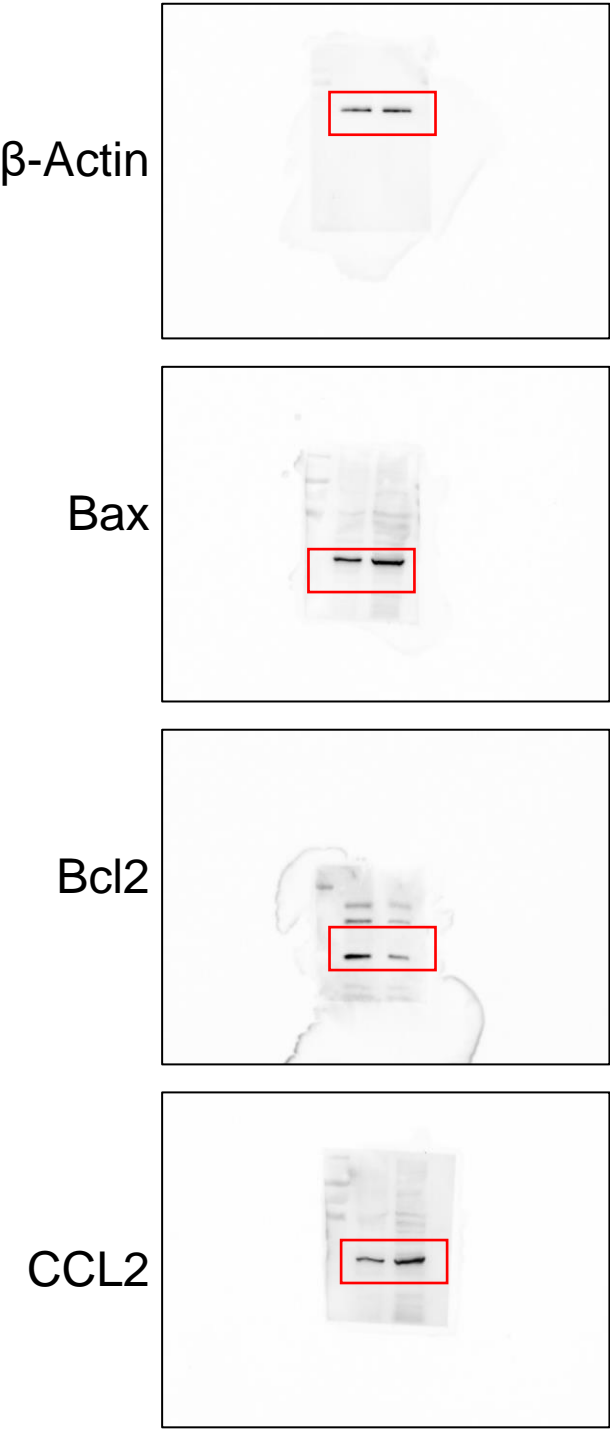

FIGURE.S1

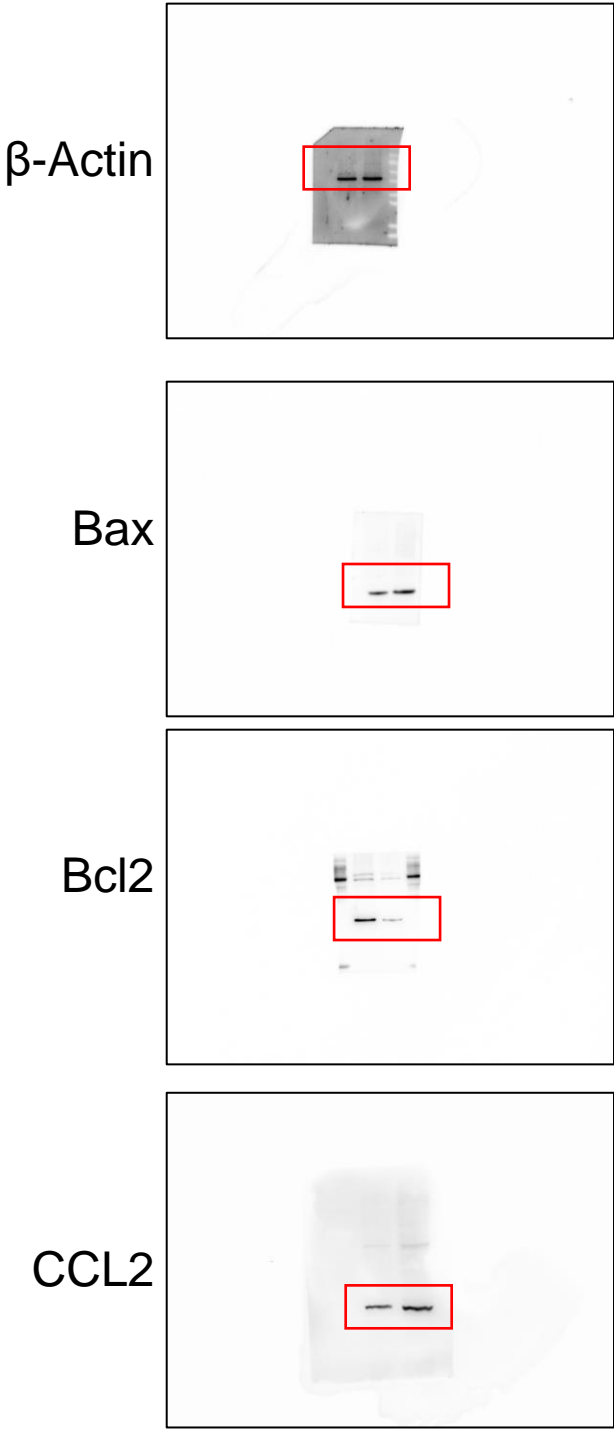

Supplement: Supplementary file 2 [file DataSheet1.pdf]

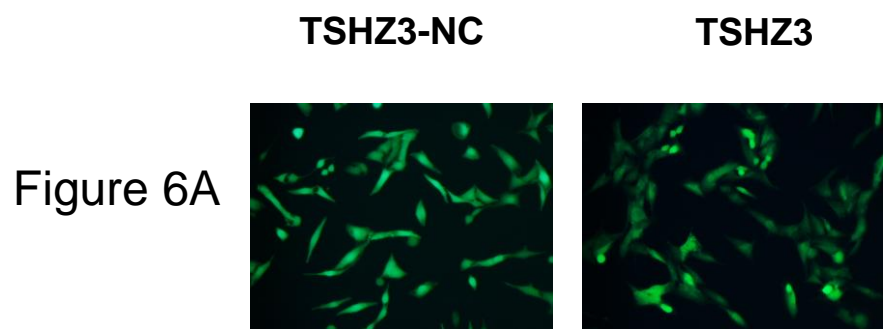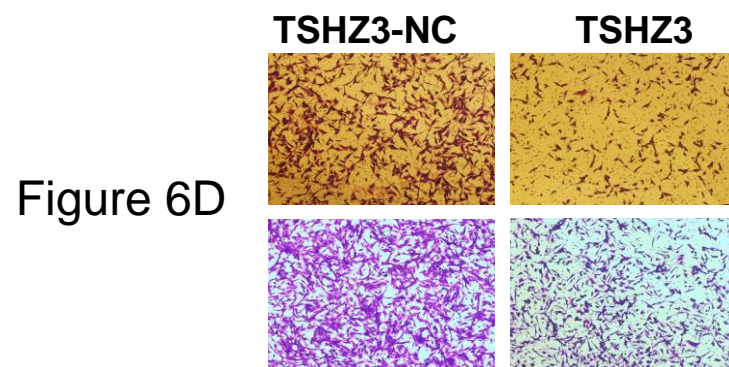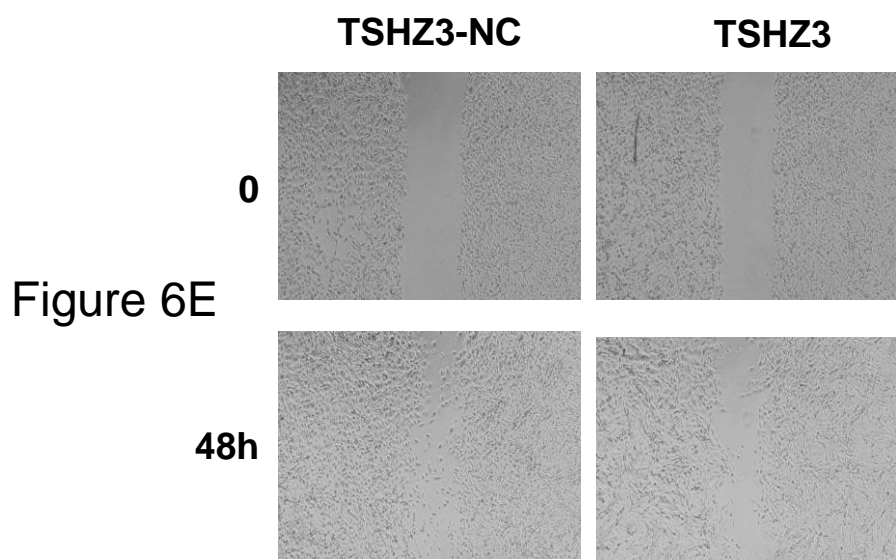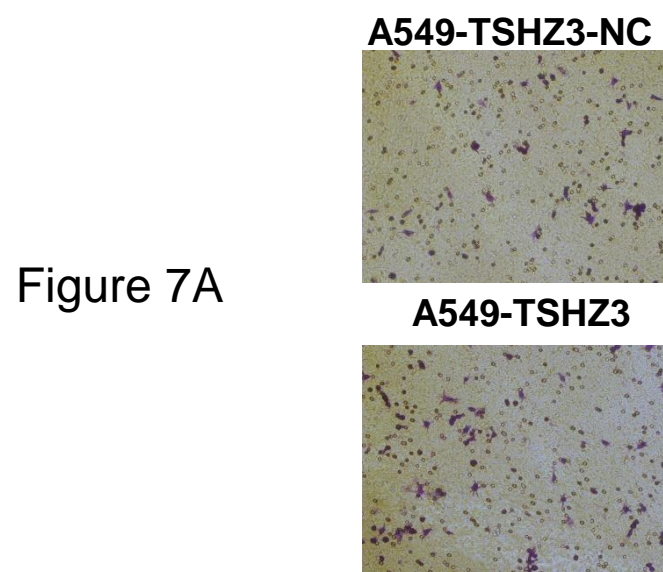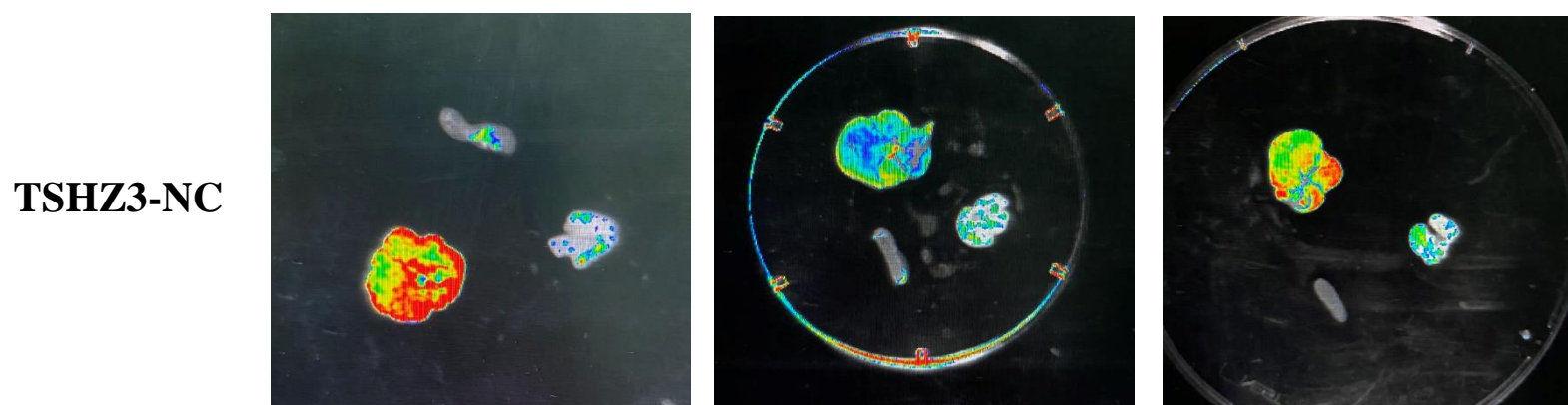

Figure 8A

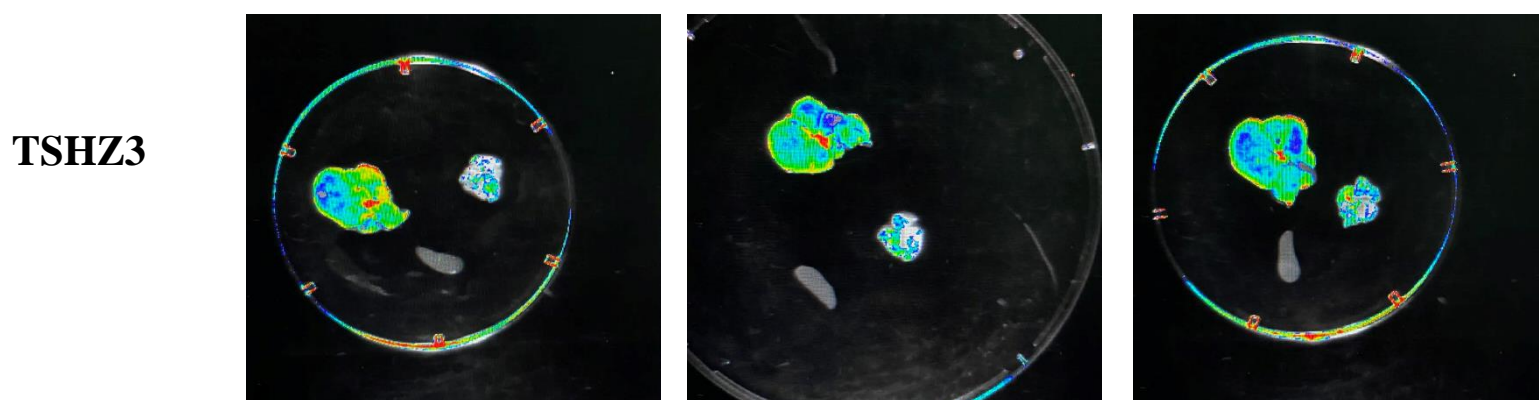

**TSHZ3-NC**

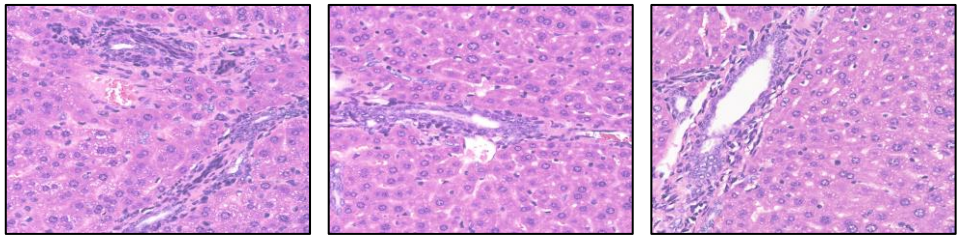

Figure 8B

**TSHZ3**

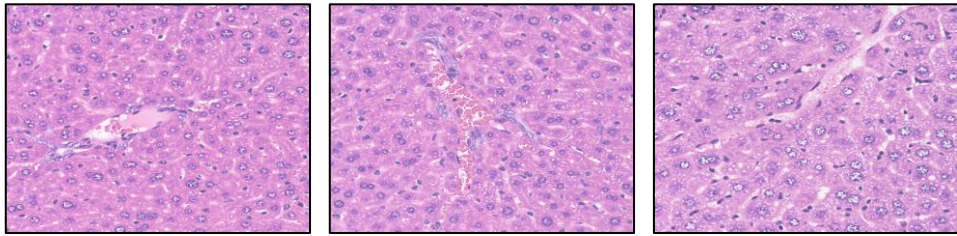

**TSHZ3-NC**

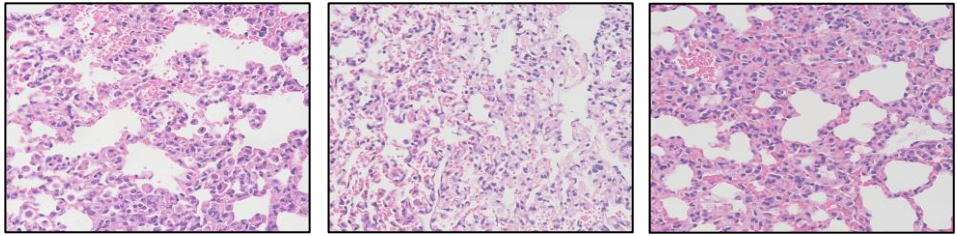

Figure 8C

**TSHZ3**

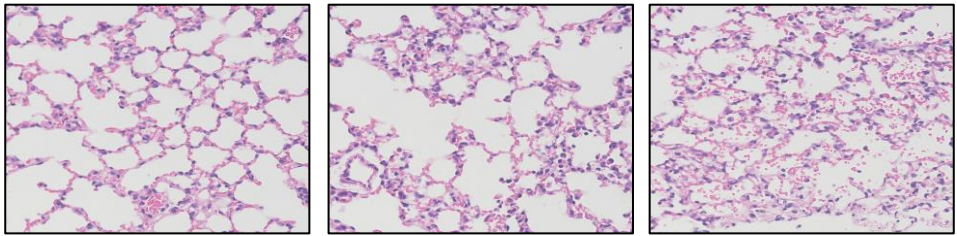

**A549-TSHZ3-NC**

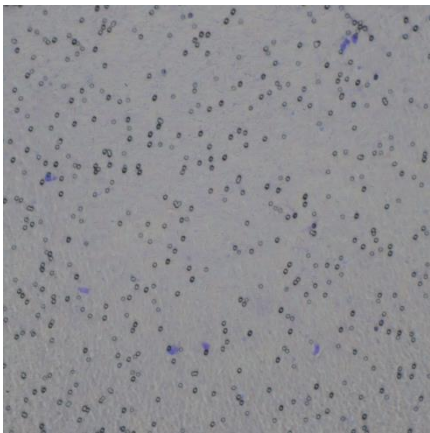

Supplementary Figure 1A

**A549-TSHZ3**

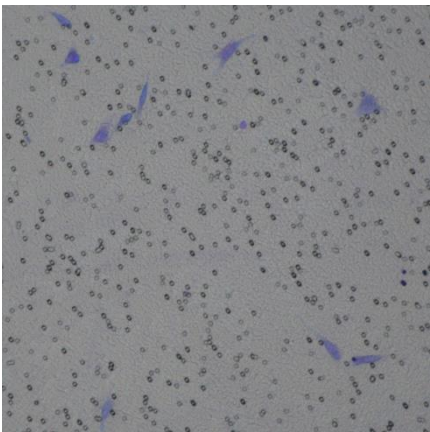

Supplement: Supplementary file 3 [file DataSheet2.pdf]
